# Remarks on Degenerative Disease, or Atrophy, of the Gastric Tubules

**Published:** 1871-02

**Authors:** Austin Flint

**Affiliations:** Professor of the Principles and Practice of Medicine and of Clinical Medicine in the Bellevue Hospital Medical College


### Selections.

### REMARKS ON DEGENERATIVE DISEASE, OR ATROPHY, OF THE GASTRIC TUBULES.

By AUSTIN FLINT, M.D., Professor of the Principles and Practice of Medicine and of Clinical Medicine in the Bellevue Hospital Medical College.

I have borne in mind my promise, made some months since, to contribute an article for the *American Practitioner* at about this date. In the fulfilment of this promise I propose to submit some remarks on degenerative disease, or atrophy, of the gastric tubules. I am led to select this subject from having read an article by Samuel Fenwick, M.D., in the London *Lancet.* The article appears in the number of that journal issued July 16, 1870; but it has not fallen under my notice until the date of my writing (December 3, 1870). In that article Dr. Fenwick reports a case in which the diagnosis of atrophy of the gastric tubules was made from the history and symptoms. The autopsy confirmed the correctness of the diagnosis. The account of the case is as follows:

"A gentleman, about forty-five years of age, consulted me in February last. He complained of great weakness, and inability for mental or bodily exertion. Occasionally he had pain in the back and a sensation of numbness in the legs; but there was no loss of feeling or appearance of paralysis. He was troubled with palpitation and breathlessness on exertion. He did not seem to be much emaciated, but his face was of the pale yellowish color so often seen in persons affected with malignant disease; and the lips, tongue, and throat were exceedingly bloodless. He had neither cough nor expectoration; the appetite was very bad; he suffered from flatulence, and occasionally from bilious vomitings, and the bowels were much confined; the pulse was exceedingly small and feeble. The complaint had come on so gradually that he could scarcely fix the exact time of its commencement; but he had been ailing for at least eighteen months. Previous to this date he had enjoyed good health; he had never suffered from any loss of blood, nor from ague or diarrhœa. On careful examination no darkness could be detected by auscultation or percussion; the liver and spleen were normal in size, shape, and position; the thyroid and lymphatic glands were not enlarged; the stomach was not dilated, and no tumor could be found in any part of the body. The urine was clear, acid, and free from albumen and sugar. A drop of blood obtained from a prick of the finger, when examined by the microscope, showed no increase, but rather a deficiency, in the number of white corpuscles. I prescribed steel and quinine, with a moderate allowance of wine."

Subsequently vomiting was a prominent and persistent symptom. The pulse became extremely feeble. There was loss of appetite and flatulency. He gradually became more and more feeble and anæmic, and after slight attacks of delirium died from exhaustion. The grounds for the diagnosis are stated in the following quotation:

"It was evident that all the symptoms from which the patient suffered arose from anæmia. But as he had been affected with no disease—such as ague, dysentery, or hemorrhage—capable of directly producing this condition, and as there was no evidence of disease of any of the viscera, it could only be supposed that there was some imperfection in the blood-making organs, or in those connected with absorption. The absence of emaciation was sufficient to prove that the powers of absorption, and of the digestion of fat and starch, were not impaired. We had therefore only to examine the condition of the organs whose office it is to digest and prepare the albuminous materials of the food—namely, the stomach and ductless glands. There was no evidence of any affection of the spleen, thyroid, thymus, or lymphatic gland; and the absence of any dark discoloration of the skin seemed to negative the supposition that the supra-renal capsules were diseased. I therefore concluded that the stomach must be the organ in fault; and as atrophy is its only morbid condition which is not accompanied by characteristic local symptoms, I diagnosed atrophy of the gland-structure as the only disease present in the case."

The examination after death showed no important changes elsewhere than in the stomach. Here the appearances were as follows:

"The stomach was empty, excepting a small quantity of gas, and its surface showed no signs of *post mortem* digestion. When placed beneath the microscope, the pits on the surface of the gastric mucuous membrane were seen to be well defined, and were rather larger than usual. The whole of the glandular structure of the organ was in a state of atrophy; in no part could I succeed in procuring a section of normal tissue. In the pyloric and middle regions the secreting tubes seemed to be converted into a mass of connective tissue; and it was only near the cardiac end that a trace of gland-structure could be observed. In this situation the gastric tubes were represented by scattered flesh-like bodies, filled with glanular matter and fatty epithelial cells. In other places the ends of the tubes were expanded into the form of cysts. Each of these was surrounded by fibres, and was lined internally by a layer of cells, the contents consisting of fat-cells and granular matter."*

The accuracy of the observation of these appearances was confirmed by Dr. Handfield Jones.

An infusion of the gastric mucous membrane, mixed with dilute hydrochloric acid, will usually, as Dr. Fenwick states, dissolve the white of egg and other albuminous substances. The experiment was made with an infusion of the mucous membrane in this case, and almost no effect was produced. Quoting the writer's words, "this experiment therefore confirmed the conclusion, derived from microscopical examination, that the gland-structure of the stomach had been so seriously diseased that it could not have been capable of performing its functions during life."

Dr. Fenwick expresses the belief that in cases of "idiopathic anæmia," described by Dr. Addison, the fatal lesions are in the gastric tubules.

The article of Dr. Fenwick is to me exceedingly interesting. Since I became acquainted with the researches of Handfield

*The morbid appearnaces are illustrated by three figures in Dr. Fenwick's article.

Jones, published in the Medico-Chirurgical Transactions, Vol. XXXVI, (London, 1854), I have entertained a strong conviction that morbid changes taking place in the gastric and intestinal tubules would be proven to enter frequently and largely into cases of disease characterized by anorexia, impaired digestion, impoverishment of the blood, and defective nutrition. For many years my attention has from time to time been directed to cases analogous to the case reported by Dr. Fenwick. I have often been disposed to report cases of this kind which have fallen under my observation. In some of these cases I have stated as the probable diagnosis degenerative lesions of the gastric tubules. It has, however, never been in my power to verify this diagnosis by microscopical examinations after death. I mean that in none of the cases to which I refer were the stomach and intestines subjected to examination with the microscope. I have repeatedly suggested to some of my friends who are occupied with microscopical researches to devote attention to the study of the glands which secrete the gastric and the intestinal juice. My friend Dr. Janeway, one of the curators of the Bellevue Hospital, distinguished for his zeal and ability in morbid anatomy, will, if this communication should meet his eye, recollect my urging him to develop knowledge and reap distinction by studying the lesions of these glands in connection with the *ante mortem* histories. Reasoning from analogy, it has seemed to me highly improbable that these glands should be exempt from the morbid changes which are known to occur in other glandular structures. This view I have presented in a brief section devoted to the subject in my work on Practice; and for more than ten years I have never failed to enlarge upon the subject in didactic teaching. I do not, of course, presume that it is of interest or importance for the profession to know how long and how much my thoughts have had this direction; but I am led to write thus in order that it may deemed not a sudden but a well matured conviction when I express a confident expectation of lesions of the gastric and intestinal glands being found hereafter to be of not less importance in pathology and clinical medicine than the renal lesions, our knowledge of which was opened up by the researches of Bright.

Are not the facts which are set forth in Dr. Fenwick's article sufficient now to warrant the nosological recognition of degeneration of the gastric tubules as a distinct affection; and as such is it not, with our present knowledge, an affection which may with much certainty be diagnosticated? Is it not probable that it may co-exist with various affections as a complication of great importance in relation to the interpretation of symptomatic phenomena and to prognosis? I do not propose to discuss these questions in this communication; but I submit them for the reflection of the readers of the *Practitioner*, with an intention of giving a further account of my own thoughts, together with my clinical observations, at some future time.

As this is an epistolary communication, may I ask you and the readers of your journal to excuse an apparent egotism in what has been written; and also in subjoining an extract from a clinical lecture on anæmia which I delivered at the Long Island College Hospital, and which was published in the *American Medical Times*, number for September, 1860? I have italicised several sentences to which I should like particulary to direct the attention of the reader:

"Before proceeding to speak of the treatment of anæmia, I wish to call attention to a form in which this condition is met with, differing from the ordinary form in this important respect: it proceeds steadily and surely to a fatal issue. I refer to the form which Addison distinguishes as 'idiopathic anæmia.' He thus distinguishes it because the anæmia is developed and continues without any adequate cause or causes being apparent. It is in these cases of anæmia that Addison was led to observe disease of the supra-renal capsules. The bronzed hue of the skin occurs in a certain proportion only of these cases. It is with the idopathic fatal anæmia, not the coloration of the skin only, that Addison supposes the disease of the supra-renal capsules to be connected. As there is, I believe, generally an incorrect impression on this point, I beg leave to read the following quotation from Addison's paper:

"'For a long period I have, from time to time, met with a very remarkable form of general anæmia, occurring without any discoverable cause whatever; cases in which there had been no previous loss of blood, no exhausting diarrhea, no chlorosis, no purpura, no renal affection, splenic miasmata, glandular strumous or malignant disease. * * * * * It was while seeking in vain to throw some additional light on this form of anæmia that I stumbled on the curious facts which it is my more immediate object just now to make known to the profession.'*

"I do not propose to discuss in this connection the question as to the existence of a pathological relation between disease

*These quotations are from a paper by Dr. Wilks in Guy's Hospital Reports, 3d series, vol. v, 1859.

of the supra-renal capsules and fatal anæmia, with or without bronzing of the skin. I will simply say I suppose it to have been shown that if there be a relation it is only occasional, not constant.

"I had myself a patient last winter who died with 'idiopathic anæmia,' together with an almost universal and a strongly-marked bronzing of the surface; and after death there was found to be no appreciable degeneration of structure in the supra-renal capsules. But not to be diverted from the point to which I wish to direct your attention, cases such as Addison refers to must have fallen under the observation of most clinical observers. I met with two or three in my hospital wards in New Orleans during the winter of 1858–9, and again during the last winter. The patients entered with marked anæmia, and greatly prostrated. Interrogation of the important organs of the body showed no serious disease. Loss of appetite was a prominent symptom, and this ended in complete anorexia. Diarrhea was more or less prominent before death; but this was not due to intestinal lesions, as was shown by examinations after death. Death took place by slow asthenia or inanition. No disease sufficient to cause death was discovered *post mortem.* Notwithstanding Addison's researches, these cases must be considered not less inexplicable now than before he stumbled on lesions in the supra-renal capsules. Even were these constant they would hardly shed much light on the occurrence of fatal anæmia. *I have not the presumption to offer an explanation of these cases; but I have an idea, which I do not hesitate to throw out because I can do no more, and I give it only for what it is worth. To follow it out by researches, which will show it to be valuable or worthless, will probably not be within my power. I suspect that in these cases there exists degenerative disease of the glandular tubules of the stomach.* I am led to this suspicion somewhat as a physician is said once to have arrived at the conclusion that the pancreas must be diseased by convincing himself that all the other organs in the body were sound; and this was the only organ which he could not satisfactorily interrogate! Here is at all events a field of research which has as yet been hardly more than explored.

"Dr. Handfield Jones is the only one within my knowledge who has made degenerative disease of the gastric tubules the subject of any investigation. He published some years ago the results of the examination, by means of the microscope, of one hundred stomachs. In seventy-seven of this number there was found more or less atrophy of the tubules. In fourteen cases the degeneration was considerable. We can as readily understand that these important organs should undergo degenerative ~~disease~~ not rendered distinctly apparent to the naked eye as that this should be true of the convoluted tubes of the kidneys. *It is perhaps the lot of some one to bring the microscope to bear upon investigations here with as much effect as Dr. Geo. Johnson has done with respect to the renal organs.* Of the importance of the stomach glands we can form an estimate when we consider that their business is to furnish from fifteen to thirty pounds of gastric juice during the twenty-four hours. Nor is it difficult to see how fatal anæmia must follow an amount of degenerative disease reducing the amount of gastric juice so far that the assimilation of food is rendered wholly inadequate to the wants of the body. *I shall be ready to claim the merit of this idea when the difficult and laborious researches of some one have shown it to be correct."—American Practitioner.*

